# LncRNA PVT1 accelerates malignant phenotypes of bladder cancer cells by modulating miR-194-5p/BCLAF1 axis as a ceRNA

**DOI:** 10.18632/aging.202203

**Published:** 2020-11-16

**Authors:** Mingwei Chen, Rongyuan Zhang, Le Lu, Jian Du, Chunyang Chen, Keke Ding, Xuedong Wei, Guangbo Zhang, Yuhua Huang, Jianquan Hou

**Affiliations:** 1 Department of Urology, The First Affiliated Hospital of Soochow University, Suzhou 215006, Jiangsu Province, China; 2 Department of Urology, The Fourth Affiliated Hospital, Zhejiang University School of Medicine, Yiwu 322000, Zhejiang Province, China; 3 Jiangsu Institute of Clinical Immunology, The First Affiliated Hospital of Soochow University, Jiangsu Key Laboratory of Clinical Immunology, Soochow University, Jiangsu Key Laboratory of Gastrointestinal Tumor Immunology, Suzhou 215006, Jiangsu Province, China

**Keywords:** PVT1, miR-194-5p, ceRNA, BCLAF1, bladder carcinoma

## Abstract

Background: Numerous studies proved that long non-coding RNA (lncRNA) is involved in the progression of multifarious diseases, especially in some carcinomas. As a potential tumor biomarker, plasmacytoma variant translocation 1 gene (PVT1) is involved in the development and progression of multifarious cancers. Nevertheless, the intrinsic and concrete molecular mechanism of PVT1 in bladder cancer still remained unclear, which is also the dilemma faced in many non-coding RNA studies.

Results: Our research revealed that PVT1 was significantly higher expression in bladder carcinoma specimens and cell lines. Further experiments indicated that knockdown or overexpression of PVT1 restrained or promoted the malignant phenotype and WNT/β-catenin signaling in bladder cancer cells. Meanwhile miR-194-5p was in contrast and miR-194-5p could partially reverse the function of PVT1 in malignant bladder tumor cells. As a microRNA sponge, PVT1 actively promotes the expression of b-cells lymphoma-2-associated transcription factor 1 (BCLAF1) to sponge miR-194-5p and subsequently increases malignant phenotypes of bladder cancer cells. Therefore, it performs a carcinogenic effect and miR-194-5p as the opposite function, and serves as an antioncogene in the bladder carcinomas pathogenesis.

Conclusion: PVT1-miR-194-5p-BCLAF1 axis is involved in the malignant progression and development of bladder carcinomas. Experiments revealed that PVT1 has a significant regulatory effect on bladder cancer (BC) and can be used as a clinical diagnostic marker and a therapeutic molecular marker for patients suffering from BC.

Methods: In urothelial bladder carcinoma specimens and cell lines, the relative expression levels of PVT1 and miR-194-5p were detected by quantitative reverse transcription PCR (RT-qPCR). Through experiments such as loss-function and over-expression, the biological effects of PVT1 and miR-194-5p on the proliferation, migration, apoptosis and tumorigenicity were explored in bladder cancer cells. Co-immunoprecipitation, proteomics experiments, dual luciferase reporter gene analysis, western blot and other methods were adopted to investigate the PVT1 potential mechanism in bladder carcinomas.

## INTRODUCTION

As the premier malignant neoplasm in the urinary system, BC is a highly pathogenic malignant tumor that puts a heavy financial strain on patients and society [1–4]. BCs frequently recurs and even advanced malignant tumors often leads to the death of BC patients, so far it is difficult to be completely cured. Therefore, it is necessary to detect the potential mechanism of BC, so as to clarify its biological function in the occurrence and development of BC, and thus it can be transformed into a reasonable treatment strategy [5–7].

At present, the treatment for BC is still limited. Most BC pathological phenotypes are urothelial carcinomas, and the relatively ideal prognosis and high morbidity non-muscle-invasive BCs account for the majority, inversely, poor prognosis is the muscle-invasive BC [2, 8–10]. The non-invasive high-risk BC is used for transurethral cystectomy (TURBT), and the radical cystectomy is mainly applicable to muscular invasive high-risk bladder carcinomas, and the advanced tumors patients could mainly adopt targeted therapy or other conservative treatment measures [11–13]. However, the treatment effect of advanced tumor patients is still not ideal and the survival rate is lower. Therefore, there is an urgent need for alternative therapies and the development of more effective and safer therapies for bladder carcinoma is inevitable [5, 14–16]. The key molecular mechanisms of BC is thoroughly explored to conquer the bottleneck of clinical treatment in the future.

With the high-efficiency development of significant sequencing technologies, more and more abnormal lncRNAs that are more than 200 nucleotides [17–21], as important diseases gene regulators referred to biological transcription and post-transcription behavior, were reported in multifarious malignant tumors [22–24]. Various literatures indicated that lncRNAs acted as important functions in the malignant tumors [25–27], and were involved in proliferation, migration, apoptosis, even metastasis and other biological processes [28–31]. The abnormal expression of PVT1 was reported in the lung carcinomas, osteosarcomas, squamous cell carcinomas, stomach carcinomas, liver carcinomas, colorectal carcinomas, nasopharyngeal carcinomas and so on [32–37]. *In vitro* and *in vivoo*, studies were implemented to in-depth study potential tumor progression roles [38–43]. However, the clinical transformation application and biological significance of PVT1 in BC and other tumors is still thoroughly uncertain [1–8, 41, 45–50].

MicroRNAs (miRNAs) are 18-25 nucleotides’ non-coding RNAs to regulate tumorigenesis and progression, which have been confirmed in various tumors [8, 28, 36]. MiRNAs are quite conserved in species evolution, which are found in plants, animals and fungi, and are only expressed in specific tissues and developmental stages. The tissue specificity and timing of microRNAs determine the functional specificity of tissues and cells, indicating that microRNAs act as various roles in term of the cell growth and development regulation [41, 50–54]. MiR-194-5p was reported in numerous tumors, including kidney carcinoma, colorectal cancer and glioma [55–59]. Nevertheless, the potential mechanisms of miR-194-5p in the malignant BC behaviors are thoroughly unknown. In our experiment, as a negative regulatory, miR-194-5p participated in the BC evolution.

A lot of literatures manifested lncRNAs function as miRNAs sponges [26, 28, 35–37, 56]. We reported the mutual correlation between PVT1 and miR-194-5p as miRNAs sponges in the bladder carcinomas. Our research could help us deeply broaden our horizons about the lncRNA-miRNA sponges’ expression pattern and mutual roles of PVT1 and miR-194-5p in the BC.

In our study, we discovered that PVT1 is up-regulated in BC tissues and cells and miR-194-5p is converse. The relative expression levels of PVT1 and miR-194-5p are closely correlated with histological grade and TNM stage. In our study, we elucidated that PVT1 facilitated the progression of BC and miR-194-5p is converse.

Furthermore, it was found that PVT1 functions as a ceRNA-dependent manner to sponge miR-194-5p to tightly promote BCLAF1 expression. Meanwhile, our deepen study suggests that PVT1 acts as a significant tumor regulator and PVT1-miR-194-5p-BCLAF1 axis participates in BC progression, which emerges its possibly clinical application of BC as a meritoriously clinical diagnosis and treatment strategies. Maybe, our experiments can offer a novel direction for the exploration of BC therapeutic target and provide a more valid research method to detect tumor progression and treatment strategies for the ultimate aim of the precision cancer medicine.

## RESULTS

### Up-regulated expression of PVT1 and down-regulated expression of miR-194-5p in BCs

**Figure 1:**
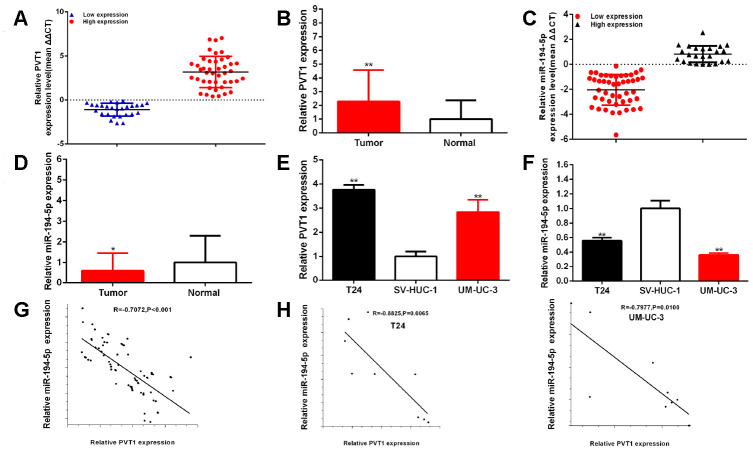
**PVT1 is up-regulated and miR-194-5p is down-regulated in BC samples and cells.** The relative expression patterns of PVT1 (**A**, **B**) and miR-194-5p (**C**, **D**) in paired BC tissues and normal tissues, and in BC cells (T24 and UM-UC-3) and SV-HUC-1 (**E**, **F**) were shown. PVT1 expression levels were statistically correlated with miR-194-5p expression levels in bladder tissues and cells (**G**, **H**). (*P < 0.05, **P < 0.01).

The relative expression level of PVT1 and miR-194-5p was measured through RT-qPCR in BC samples. Compared to para-carcinoma tissues, the relative expression level of PVT1 was significantly increased about 2.28 times (43 of 70) of BC samples (P<0.001) (Figure 1A, 1B), and the relative expression level of miR-194-5p was significantly decreased about 42.92% (46 of 70) of BC samples (P=0.023) (Figure 1C, 1D). Compared to SV-HUC-1, the relative expression level of PVT1 was up-regulated in both BC cells, T24 about 3.76 times (P < 0.001) and UM-UC-3 about 2.84 times (P=0.004) (Figure 1E), and the relative expression level of miR-194-5p was decreased in both BC cells, T24 about 44.16% (P=0.003) and UM-UC-3 about 63.77% (P = 0.001) (Figure 1F). PVT1 expression levels were statistically positively correlated with miR-194-5p expression levels in BC samples and cells (Figure 1G, 1H). As following in Table 1, the up-regulated expression of PVT1 was closely associated with histological grade (P = 0.014), and TNM stage (P< 0.001) in bladder carcinomas, the low expression of miR-194-5p was significantly related to histological grade (P=0.006) and TNM stage (P<0.001). But sex, age, tumor size, and lymph node metastasis were no obvious correlation with the relative expression levels of PVT1 and miR-194-5p. Our results manifested that PVT1 could act as the oncogene and miR-194-5p could act as the antioncogene in BCs.

**Table 1a:** Correlation between PVT1 expression and clinicopathological characteristics of bladder cancer patients.

**Characteristics**	**Total**	**Expression of PVT1**		**P value**
		**High (n=43)**	**Low (n=27)**	
Gender				
Male	45	31(68.9%)	14(31.1%)	0.124
Female	25	12(48.0%)	13(52.0%)	
Tumor size (cm)				
<3cm	33	17(51.5%)	16(48.5%)	0.142
≥3cm	37	26(70.3%)	11(29.7%)	
Multiplicity				
Single	30	20(66.7%)	10(33.3%)	0.468
Multiple	40	23(57.5%)	17(42.5%)	
Age				
<60	26	18(69.2%)	8(30.8%)	0.324
≥60	44	25(56.8%)	19(43.2%)	
Histological grade				
PUNLMP/Low-grade	33	15(45.5%)	18(54.5%)	0.014*
High-grade	37	28(75.7%)	9(24.3%)	
Tumor invasion depth (T)				
Tis, Ta, T1	34	13(38.2%)	21(61.8%)	<0.001**
T2,T3 or above	36	30(83.3%)	6(16.7%)	
Lymph node metastasis(N)				
N0	64	38(59.4%)	26(40.6%)	0.394
N1 or above	6	5(83.3%)	1(16.7%)	

PUNLMP: Papillary Urothelial Neoplasm of Low Malignant Potential; Low-grade, Low-grade Papillary Urothelial Carcinoma; High-grade, High-grade Papillary Urothelial Carcinoma; TNM according to the seventh edition of staging TNM of Union Internationale Contre Le Cancer (UICC) in 2009.

**Table 1b:** Correlation between miR-194-5p expression and clinicopathological characteristics of bladder cancer patients.

**Characteristics**	**Total**	**Expression of miR-194-5p**		**P value**
		**High (n=24)**	**Low (n=46)**	
Gender				
Male	45	13(28.9%)	32(71.1%)	0.293
Female	25	11(44.0%)	14(56.0%)	
Tumor size (cm)				
<3cm	33	13(39.4%)	20(60.6%)	0.455
≥3cm	37	11(29.7%)	26(70.3%)	
Multiplicity				
Single	30	14(46.7%)	16(53.3%)	0.077
Multiple	40	10(25.0%)	30(75.0%)	
Age				
<60	26	12(46.2%)	14(53.8%)	0.125
≥60	44	12(27.3%)	32(72.7%)	
Histological grade				
PUNLMP/Low-grade	33	17(51.5%)	16(48.5%)	0.006**
High-grade	37	7(18.9%)	30(81.1%)	
Tumor invasion depth (T)				
Tis, Ta, T1	34	21(61.8%)	13(38.2%)	<0.001**
T2,T3 or above	36	3(8.30%)	33(91.7%)	
Lymph node metastasis(N)				
N0	64	23(35.9%)	41(64.1%)	0.656
N1 or above	6	1(16.7%)	5(83.3%)	

PUNLMP: Papillary Urothelial Neoplasm of Low Malignant Potential; Low-grade, Low-grade Papillary Urothelial Carcinoma; High-grade, High-grade Papillary Urothelial Carcinoma; TNM according to the seventh edition of staging TNM of Union Internationale Contre Le Cancer (UICC) in 2009.

### PVT1 acted as the oncogene

**Figure 2:**
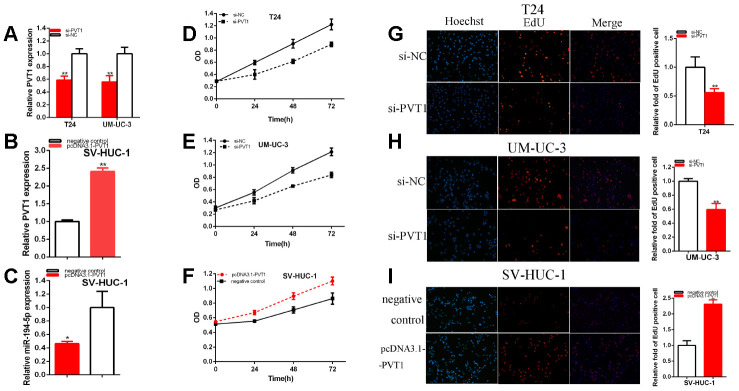
**PVT1 acted as the oncogene.** The relative expression level of PVT1 was reduced by si-PVT1 (**A**) and increased by pcDNA3.1-PVT1 (**B**). The relative expression level of miR-194-5p was reduced by pcDNA3.1-PVT1 (**C**). Cell proliferation was detected in both BC cells after transfection of siRNA (**D**, **E**) and pcDNA3.1-PVT1 (**F**). Representative images of EdU assay and the relative fold changes of EdU positive cells were detected by siRNA (**G**, **H**) and pcDNA3.1-PVT1 (**I**). (*P < 0.05, **P < 0.01).

After 48 hours of transfection for siRNA, miRNA mimics or inhibitor and pcDNA3.1-PVT1, the relative expression levels of PVT1 and miR-194-5p were detected by qRT-PCR in T24, UM-UC-3 and SV-HUC-1. After transfection, the relative expression of PVT1 was down-regulated about 41.51% in T24 (p= 0.002) and was reduced about 44.32% in UM-UC-3 (P= 0.006) cells (Figure 2A). The relative expression of PVT1 was notably increased about 2.41 times in SV-HUC-1 (p<0.001) after 48 hours of transfection for pcDNA3.1-PVT1 (Figure 2B). The relative expression of miR-194-5p was reduced about 53.60% in SV-HUC-1 (P= 0.0185) by pcDNA3.1-PVT1 (Figure 2C).

CCK-8 was carried out to detect whether the knockdown of PVT1 could restrain the proliferation and pcDNA3.1-PVT1 could facilitate the proliferation in BC T24, UM-UC-3 and SV-HUC-1 cells. Our study demonstrated that si-PVT1 (Figure 2D, 2E) notably restrained both BC cells proliferation (p < 0.01). PcDNA3.1-PVT1 (Figure 2F) remarkably facilitated SV-HUC-1 cell proliferation (p < 0.01).

EdU was elucidated cell proliferation as well. Compared with control group, EdU positive T24 and UM-UC-3 cells in si-PVT1 group were reduced and pcDNA3.1-PVT1 group was reversed after transfection in SV-HUC-1 cell line.

EdU assay proved that the quantity of EdU positive cells was decreased about 47.98% in T24 (P=0.006) (Figure 2G) and about 40.53% in UM-UC-3 (P = 0.002) (Figure 2H) in si-PVT1 group. The quantity of EdU positive cells was increased about 2.31 times in SV-HUC-1 (P< 0.001) (Figure 2I) of pcDNA3.1-PVT1 group.

Our study manifested that the knockdown of PVT1 restrained bladder cell lines proliferation and over-expression PVT1 facilitated bladder cell lines proliferation.

**Figure 3:**
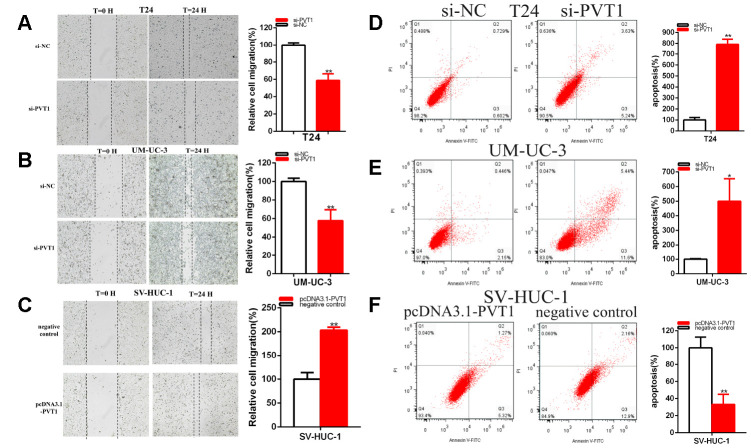
**PVT1 affected the BC cell migration and apoptosis.** The relative cell migration was restrained after transfection of siRNA in the T24 and UM-UC-3 (**A**, **B**) cell lines. The relative cell migration was facilitated after transfection of pcDNA3.1-PVT1 in the SV-HUC-1 cell lines (**C**). Apoptotic cells were measured after transfection of siRNA in the T24 (**D**) and UM-UC-3 (**E**) cell lines, of pcDNA3.1 in the SV-HUC-1 cell line (**F**) by flow cytometry analysis. (*P < 0.05, **P < 0.01).

Cell migration was detected after the transfection of siRNA and plasmids by scratch assay. Scratch assay that revealed that the ratio of the relative migration was decreased about 41.29% in T24 (P = 0.001) (Figure 3A) and decreased about 42.18% in UM-UC-3 (P = 0.004) (Figure 3B) in si-PVT1 group. The ratio of the relative migration in the pcDNA3.1-PVT1 group was up-regulated about 2.032 times in SV-HUC-1 (P<0.001) (Figure 3C).

Our results concluded that the knockdown of PVT1 restrained bladder cell lines migration. Overexpression PVT1 expression facilitated bladder cell lines migration.

Cell apoptosis was measured with flow cytometry assay. Compared with control groups, the ratios of apoptosis were up-regulated about 7.91 times in T24 (P<0.001) and increased about 4.99 times in UM-UC-3 (P=0.011) (Figure 3D, 3E) after transfection si-PVT1.

Compared with control groups, the ratios of apoptosis were dramatically reduced about 0.332 times in SV-HUC-1 (P=0.003) (Figure 3F) after the transfection of pcDNA3.1-PVT1.

In brief, the knockdown of PVT1 facilitated bladder cell lines apoptosis and overexpression PVT1 restrained bladder cell lines apoptosis.

### MiR-194-5p acted as the antioncogene

**Figure 4:**
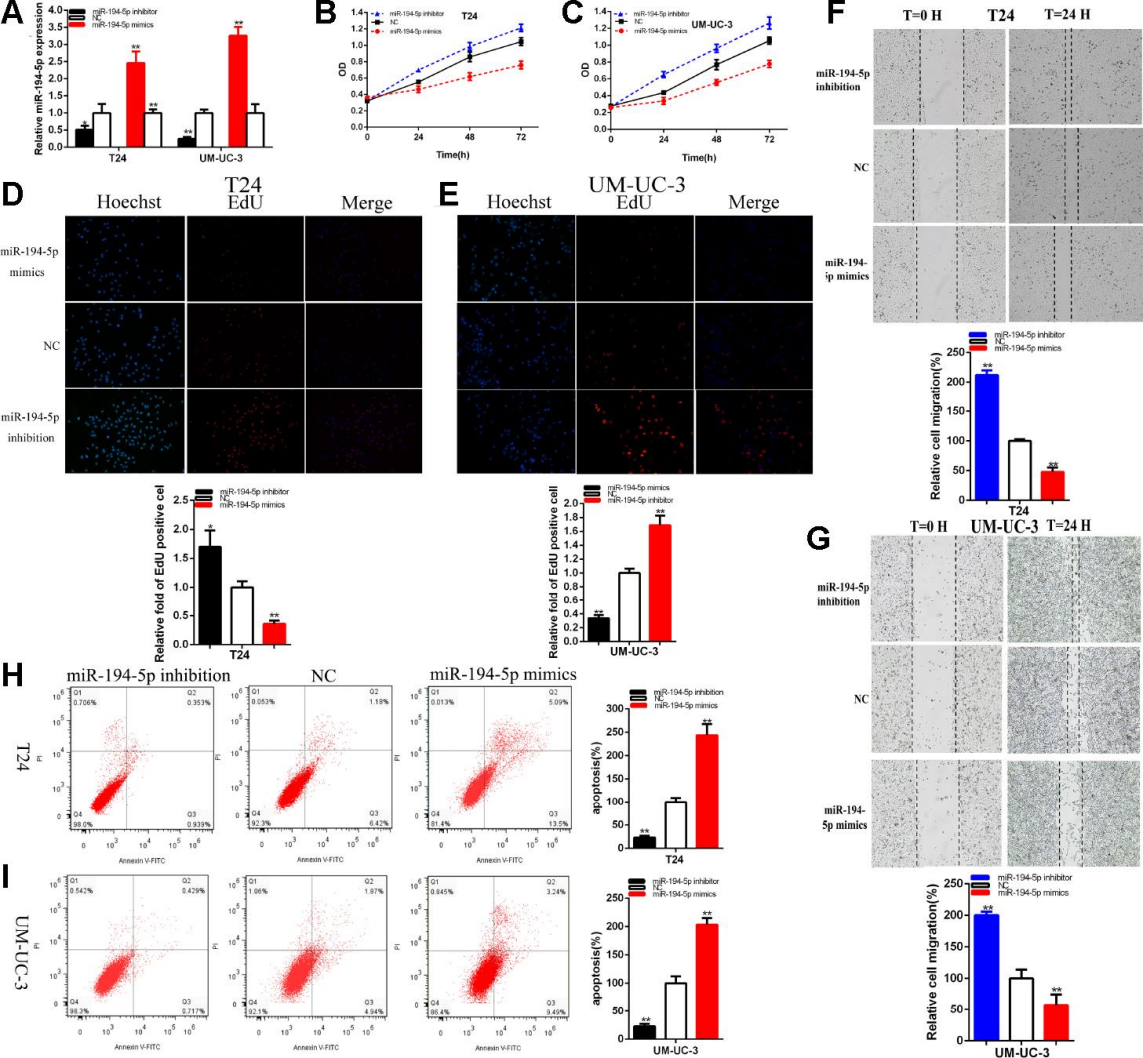
**MiR-194-5p acted as the antioncogene.** The relative expression level of miR-194-5p was decreased by miR-194-5p inhibitor and increased by miR-194-5p mimics (**A**). Cell proliferation was detected in both BC cells after the transfection of miR-194-5p inhibitor or mimics (**B**, **C**). Representative images of EdU assay and the relative fold changes of EdU positive cells were detected by miR-194-5p inhibitor or mimics (**D**, **E**). The relative cell migration was restrained or accelerated after the transfection of miR-194-5p mimics or inhibitor in the T24 and UM-UC-3 (**F**, **G**) cell lines. Apoptotic cells were measured after the transfection of miR-194-5p mimics or inhibitor in the T24 and UM-UC-3(**H**, **I**) cell lines by flow cytometry analysis. (*P < 0.05, **P < 0.01).

The relative expression levels of miR-194-5p were remarkably reduced about 48.64% in T24 (p =0.044) and about 75.51% in UM-UC-3 (P<0.001) at 48 hours after the transfection of miR-194-5p inhibitor (Figure 4A). The relative expression levels of miR-194-5p were increased in about 2.460 times in T24 (p=0.002) and 3.259 times in UM-UC-3 (p< 0.001) at 48 hours after the transfection of miR-194-5p mimics (Figure 4A).

The results demonstrated that miR-194-5p mimics significantly restrained both BC cells proliferation remarkably (Figure 4B, 4C) (p < 0.01). MiR-194-5p inhibitor accelerated both BC cells proliferation (Figure 4B, 4C) (p < 0.01).

The quantity of Edu positive cells in miR-194-5p mimics group was notably decreased about 64.34% in T24 (P= 0.001) and dramatically reduced about 66.34% in UM-UC-3 (P< 0.001) (Figure 4D, 4E). The quantity of Edu positive cells in miR-194-5p inhibitor group was up-regulated about 1.70 times in T24 (P= 0.015) and elevated about 1.69 times in UM-UC-3 (P= 0.001) (Figure 4D, 4E). Our study illustrated that the up-regulation of miR-194-5p restrained BC cells proliferation and suppression of miR-194-5p accelerated BC cells proliferation.

The ratio of the relative migration was down-regulated about 52.10% in T24 (P< 0.001) and decreased about 42.53% in UM-UC-3 (P=0.0002) (Figure 4F, 4G) after the transfection of miR-194-5p mimics. The ratio of the relative migration was up-regulated about 2.121 times in T24 (P< 0.001) and increased about 2.002 times in UM-UC-3 (P=0.0003) (Figure 4F, 4G) after transfection miR-194-5p inhibitor. These data suggested that over-expression of miR-194-5p suppressed BC cells migration and knockdown of mi-194-5p accelerated BC cells migration.

Compared to NC groups, the ratios of apoptosis were gone up about 2.434 times in T24 (P=0.0001) and increased about 2.033 times in UM-UC-3 (P=0.0004) (Figure 4H, 4I) after transfection miR-194-5p mimics. Compared to NC groups, the ratios of apoptosis were lessened about 0.230 times in T24 (P= 0.0002) and about 0.228 times in UM-UC-3 (P=0.0005) (Figure 4H, 4I) after transfection miR-194-5p inhibitor. Ultimately, over-expression of miR-194-5p accelerated BC cells apoptosis and down-regulation of mi-194-5p restrained BC cells apoptosis.

### PVT1 sponges miR-194-5p

**Figure 5:**
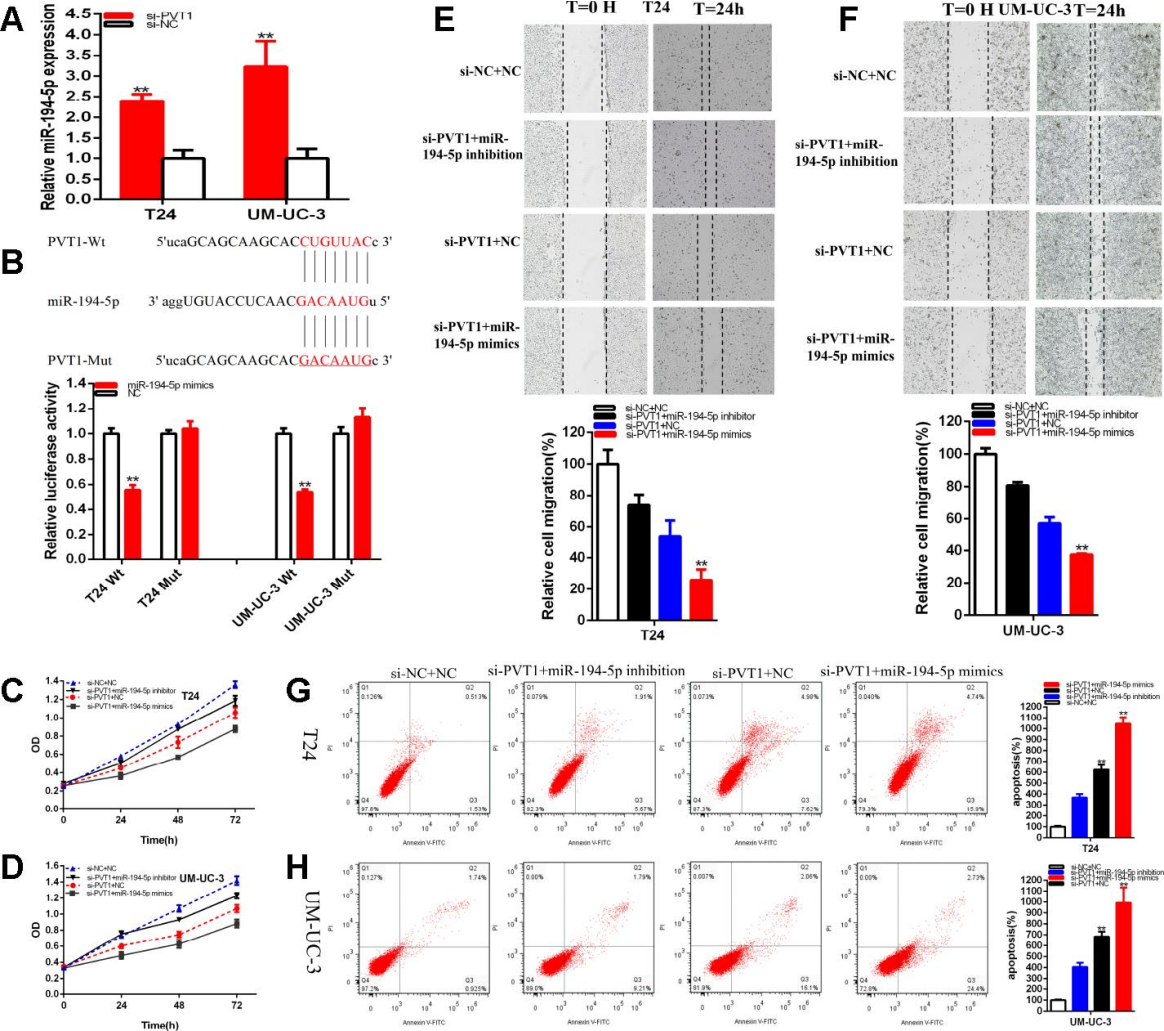
**PVT1 was a target of miR-194-5p.** The relative expression of miR-194-5p was increased by si-PVT1 (**A**). Dual-luciferase reporter assays were performed in T24 or UM-UC-3 cells co-transfected with PVT1-Wt or PVT1-Mut and miR-194-5p mimics or NC (**B**). Cell proliferation was detected in both BC cell lines after co-transfection with si-NC+NC, si-PVT1+miR-194-5p inhibitor or si-PVT1+miR-194-5p mimics (**C**, **D**). The relative cell migration after the co-transfection with si-NC+NC, si-PVT1+miR-194-5p inhibitor or si-PVT1+miR-194-5p mimics, and the representative images were as follow (**E**, **F**). The apoptotic cells were measured after the co-transfection with si-C+NC, si-PVT1+miR-194-5p inhibitor or si-PVT1+miR-194-5p mimics by flow cytometry analysis (**G**, **H**). (*P < 0.05, **P < 0.01).

Compared with si-NC groups, the miR-194-5p expressions were increased about 2.393 times in T24 (P=0.001) and about 3.223 times in UM-UC-3 (P= 0.004) (Figure 5A) in si-PVT1 groups. We used bioinformatics databases to predict some underlying binding sites of PVT1 with miR-194-5p. We verified the predictions by luciferase reporter assay. Our study proved that miR-194-5p mimics significantly suppressed PVT1 wild type reporter luciferase activity. Compared with the co-transfections NC+ pmirGLO-PVT1-Wt, the luciferase activity was notably reduced about 44.31% in T24 (P=0.0002) and about 45.88% in UM-UC-3 (P<0.001) in the co-transfections miR-194-5p mimics + pmirGLO-PVT1-Wt, nevertheless, miR-194-5p could not suppress the PVT1 mutant reporter vector luciferase activity (Figure 5B).

### PVT1 sponging miR-194-5p mediated BC cell progression

The luciferase reporter assays have verified that PVT1 sponged miR-194-5p. Firstly, we must verify that miR-194-5p co-regulated the repressive progression induced by knockdown PVT1 in BC cells. That si-PVT1 co-transfected miR-194-5p mimics could manifest more powerfully suppressed effects on BC cells proliferation (Figure 5C, 5D) and migration (Figure 5E, 5F) than si-NC co-transfection with NC (si-NC+NC). Meanwhile, compared with si-NC+NC group, apoptosis was accelerated in si-PVT1 co-transfection miR-194-5p mimics (si-PVT1+miR-194-5p mimics) group (Figure 5G, 5H). Conversely, miR-194-5p inhibitor could partially reverse inhibited effects on BC cells progression induced by si-PVT1**.**

The CCK-8 assays have manifested that si-PVT1 co-transfected miR-194-5p mimics (Figure 5C, 5D) remarkably restrained both BC cells proliferation in (p<0.01 in T24 and UM-UC-3). At the same time, miR-194-5p inhibitor could partially reverse inhibited effects on BC cells proliferation induced by si-PVT1 (Figure 5C, 5D).

Compared with si-NC+NC, si-PVT1 co-transfected miR-194-5p mimics could decrease the ratio of the relative migration about 77.28% in T24 (P= 0.0003) and about 62.39% in UM-UC-3 (P< 0.001). Moreover, miR-194-5p inhibitor could partially reverse inhibited effects on BC cells migration induced by si-PVT1 and increased about 19.86% in T24 and about 23.55% in UM-UC-3 cell lines (Figure 5E, 5F).

Compared with si-NC+NC, si-PVT1 co-transfected miR-194-5p mimics could accelerate the ratio of the relative apoptosis about 10.44 times in T24 (P<0.001) and about 9.91 times in UM-UC-3 (P<0.001), what’s more, miR-194-5p inhibitor could partially reverse promoting apoptosis on BC cells migration induced by si-PVT1 and reduced about 255.36% in T24 and about 277.04% in UM-UC-3 (Figure 5G, 5H).

### PVT1 sponging miR-194-5p closely promoted BCLAF1 expression

**Figure 6:**
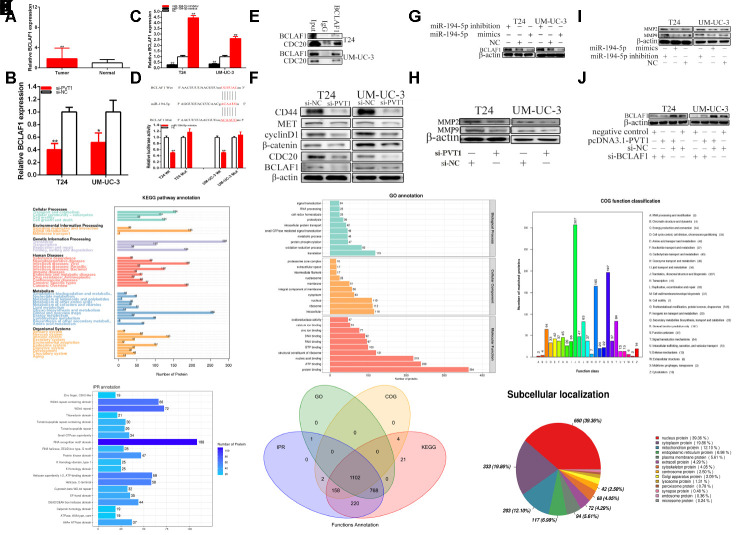
**PVT1 regulates BCLAF1 expression via sponging miR-194-5p.** The relative expression patterns of BCLAF1 were detected in paired bladder carcinomas tissues and normal tissues (**A**). The relative expression of BCLAF1 was reduced by si-PVT1 (**B**). Overexpressing miR-194-5p down-regulated BCLAF1 expression and knockdown of miR-194-5p up-regulated BCLAF1 expression in BC cells (**C**). Dual-luciferase reporter assay showed BCLAF1-Wt and miR-194-5p mimics co-transfection restrained luciferase activity (**D**). Immunoprecipitation assays were used to identify proteins associated with BCLAF1. The anti- BCLAF1 and IgG antibody were incubated with cell extracts and CDC20 was identified to be a BCLAF1-binding protein in the cancer cell line (**E**). Knockdown of PVT1 decreased BCLAF1 etc. expression in BC cells (**F**). Overexpressing miR-194-5p decreased BCLAF1 expression and knockdown of miR-194-5p increased BCLAF1 expression in BC cells (**G**). Knockdown of PVT1 decreased MPP2 and MPP9 expression in BC cells (**H**). Overexpressing miR-194-5p decreased MPP2 and MPP9 expression and knockdown of miR-194-5p increased MPP2 and MPP9 expression in BC cells (**I**). Knockdown of BCLAF1 reversed BCLAF1 expression promotion induced by overexpression PVT1 in BC cells (**J**). IP pyrolysis products are used for proteomics detection (**Ka**–**f**): a-KEGG, b-GO, c-COG, d-IPR, e-function annotation, f-subcellular localization (*P < 0.05, **P < 0.01).

Compared with para-carcinoma tissues, the relative expression level of BCLAF1 was significantly increased about 1.82 times of BC samples (P=0.002) (Figure 6A). Compared with si-NC groups, the BCLAF1 expressions were decreased about 59.97% in T24 (P=0.001) and about 48.52% in UM-UC-3 (P=0.025) (Figure 6B) in si-PVT1 groups. Compared with NC groups, the BCLAF1 expressions were decreased in T24 and UM-UC-3 (P<0.01) after the transfection of miR-194-5p mimics and were up-regulated in T24 and UM-UC-3 (P<0.01) after the transfection of miR-194-5p inhibition (Figure 6C).

We used bioinformatics databases to predict BCLAF1 with miR-194-5p possible mutual binding sites, which was shown as Figure 6D. The predicted binding sites and binding effects were manifested through luciferase reporter assay.

Compared with the co-transfections with NC + pmirGLO-PVT1-Wt, our results have confirmed that miR-194-5p mimics dramatically restrained BCLAF1 wild type reporter luciferase activity, which decreased about 49.66% in T24 (p< 0.001) and about 49.47% in UM-UC-3 (p<0.001) in the co-transfection with miR-194-5p mimics + BCLAF1C-3’UTR-Wt. Inversely, miR-194-5p mimics could not restrain the BCLAF1 mutant binding sites reporter vector luciferase activity (Figure 6D).

We identified that protein is associated with BCLAF1 through immunoprecipitation (IP/co-IP) assays. CDC20 was detected to be a BCLAF1-binding protein in the bladder cell lines (Figure 6E). At the same time, IP pyrolysis products are used for proteomics detection (Figures 6Ka–f), which was furthermore proved that BCLAF1 bound multiple functional proteins in BC cells, and KEGG, GO and other analysis were performed (Also as shown as the GO.anno,KEGG.anno and Subcellular.localization_anno), and we further revealed that BCLAF1 could interact with CDC20.

Our study proved that the relative expression level of PVT1 was closely related to BCLAF1 expression and down-regulation of PVT1 could reduce BCLAF1 expression in BC cells. Our further experiments confirmed that restrained PVT1 referred to the WNT signaling in BC cells (Figure 6F). Western blotting was used to detect the WNT signaling that is associated downstream genes expression. Knockdown of PVT1 decreased CD44, MET, cyclinD1, CDC20, BCLAF1 and β-catenin expression in BC cells. Therefore, the down-regulation of PVT1 inhibited the progression of BC cells through inhibition of the Wnt/β-catenin signaling pathway because β-catenin was the vital protein in this signaling pathway.

We must confirm whether PVT1 increased BCLAF1 expression via miR-194-5p-dependent manner in BC cells. Our studies proved that over-expression of miR-194-5p reduced BCLAF1 expression and miR-194-5p inhibition up-regulated expression of BCLAF1 in BC cells (Figure 6G). Our experiments indicated that PVT1 could closely promote BCLAF1 expression via sponging miR-194-5p in BC cells.

The knockdown of PVT1 caused the reduction of MMP-2 and MMP-9 (Figure 6H). Thus, we concluded that the knockdown of PVT1 suppressed the migration of BC cell lines, which is in accordance with that after transfection miR-194-5p inhibition and mimics. Over-expression miR-194-5p decreased MPP2 and MPP9 expression and the knockdown of miR-194-5p increased MPP2 and MPP9 expression in BC cells (Figure 6I). Therefore, we concluded that PVT1 and miR-194-5p referred to the migration of BC cell lines.

### PVT1 accelerated malignant phenotypes of BC cells via BCLAF1-dependent manner

**Figure 7:**
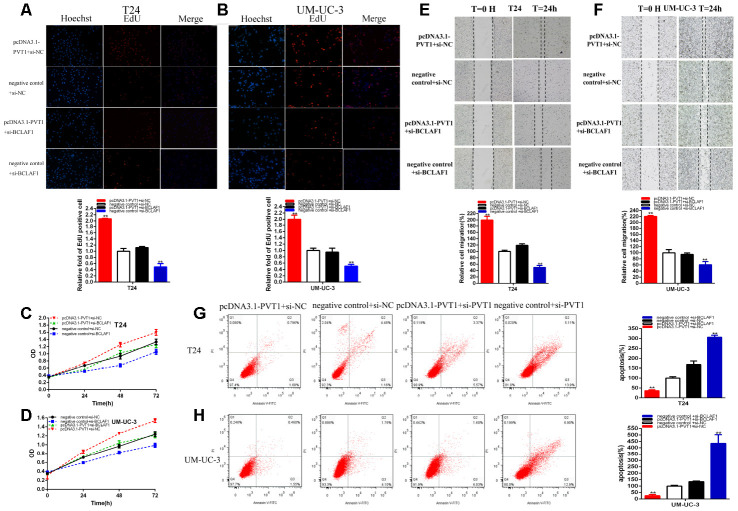
**PVT1 positively regulates BCLAF1 expression via sponging miR-194-5p.** Knockdown BCLAF1 significantly reversed cell proliferation promotion induced by Overexpression PVT1(EdU, **A**, **B**, CCK8, **C,** **D**). Knockdown BCLAF1 significantly reversed cell migration promotion induced by overexpression PVT1(**E**, **F**). Knockdown BCLAF1 significantly reversed cell apoptosis inhibition induced by overexpression PVT1 (**G**, **H**). (*P < 0.05, **P < 0.01).

We should confirm whether PVT1 accelerated malignant phenotypes via BCLAF1-dependent manner in BC cells. Further experiments proved that knockdown of BCLAF1 dramatically reversed the promotion of BCLAF1 expression induced by over-expression of PVT1 in BC cells (Figure 6J). Moreover, the knockdown of BCLAF1 reversed BC cells proliferation promotion (Figure 7A–7D) induced by over-expression of PVT1. And BCLAF1 knockdown could reverse BC cells migration (Figure 7E, 7F) induced by over-expression of PVT1. Meanwhile, knockdown of BCLAF1 could notably reverse BC cells apoptosis suppression (Figure 7G, 7H) induced by over-expression of PVT1. Our studies proved that PVT1 could accelerate malignant phenotypes of BC cells via BCLAF1-dependent manner.

**Figure 8:**
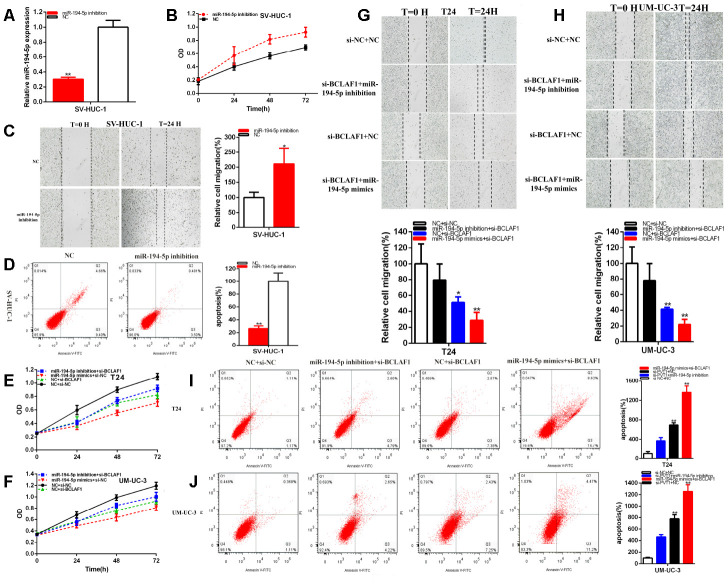
**MiR-194-5p positively regulates BCLAF1 expression.** The relative expression level of miR-194-5p was reduced by miR-194-5p inhibitor in SV-HUC-1 (**A**). Cell proliferation was detected in SV-HUC-1 after transfection of miR-194-5p inhibitor (**B**). The relative cell migration was accelerated after transfection of miR-194-5p inhibitor in the SV-HUC-1 (**C**). Apoptotic cells were measured after transfection of miR-194-5p inhibitor in SV-HUC-1 (**D**). Cell proliferation was detected in both bladder carcinomas cell lines after co-transfection with si-NC+NC and si-BCLAF1+miR-194-5p inhibitor or mimics (**E**, **F**). The relative cell migration after co-transfection with si-NC+NC, si-BCLAF1+miR-194-5p inhibitor or mimics, and the representative images were as follow (**G**, **H**). The apoptotic cells were measured after co-transfection with si-C+NC, si-BCLAF1+miR-194-5p inhibitor or mimics by flow cytometry analysis (**I**, **J**). (*P < 0.05, **P < 0.01).

The relative expression levels of miR-194-5p were significantly decreased in about 69.80% in SV-HUC-1 (p=0.0002) (Figure 8A). MiR-194-5p inhibition notably promoted bladder cells proliferation (Figure 8B) (p < 0.01). The ratio of the relative migration was up-regulated about 2.109 times in SV-HUC-1 (P= 0.026) (Figure 8C) after transfection miR-194-5p inhibitor.

These data suggested that the knockdown of mi-194-5p accelerated bladder cells migration. Compared with NC groups, the ratios of apoptosis were lessened about 0.264 times in SV-HUC-1 (P= 0.0008) (Figure 8D) after transfection miR-194-5p inhibitor.

MiR-194-5p co-regulated the repressive progression induced by knockdown BCLAF1 in BC cells. That si-BCLAF1 co-transfected miR-194-5p mimics could manifested more powerfully suppressed effects on BC cells proliferation (Figure 8E, 8F) and migration (Figure 8G, 8H) than si-NC co-transfection with NC (si-NC+NC), meanwhile, compared with si-NC+NC group, apoptosis was accelerated in si-BCLAF1 co-transfection miR-194-5p mimics (si-BCLAF1+miR-194-5p mimics) group (Figure 8I, 8J). Conversely, miR-194-5p inhibitor could partially reverse inhibited effects on BC cells progression induced by si-BCLAF1**.**

### Knockdown of PVT1 suppressed BC cells tumorigenicity

**Figure 9:**
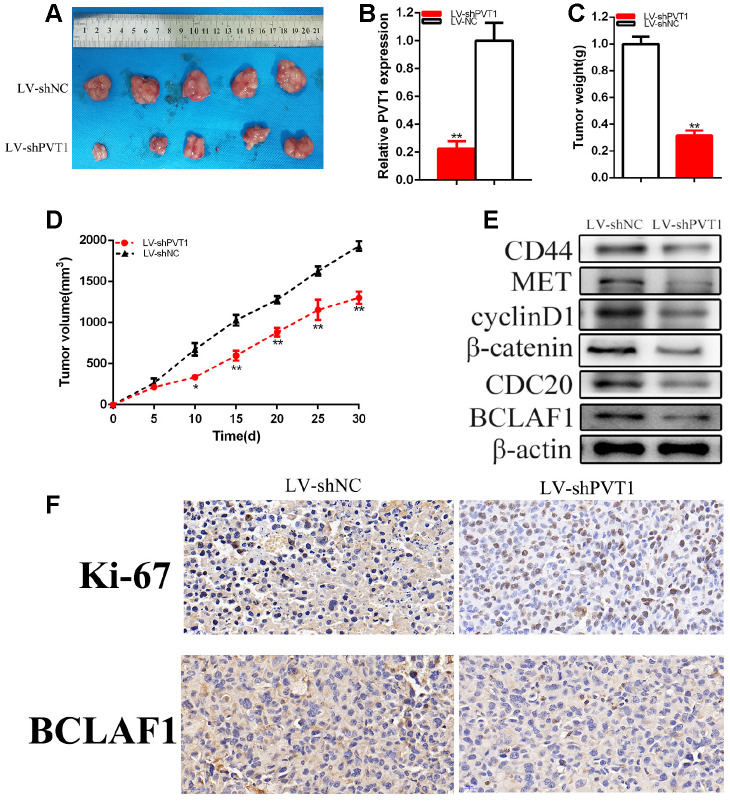
**Knockdown of PVT1 suppressed BC cells tumorigenicity.** Tumors collected from mice were exhibited (**A**). The relative expression level of PVT1 was reduced by LV-PVT1 (**B**). Tumor weight of LV-PVT1 or LV-shNC groups were measured (**C**). Tumor volume curve of LV-shPVT1 or LV-shNC groups were measured (**D**). Knockdown of PVT1 down-regulated BCLAF1 etc. expression (**E**). Knockdown of PVT1 decreased Ki-67 and BCLAF1 expression of BC cells *in vivo* (**F**). (*P < 0.05, **P < 0.01).

The generation of xenograft was used to confirm whether PVT1 promoted the tumorigenicity of BC cells. Our experiments proved that the knockdown of PVT1 could restrain the tumorigenicity of BCs *in vivo* (Figure 9A–9F). Solid tumors that were obtained from mice were displayed in Figure 9A. It was confirmed that the relative expression of PVT1 was reduced in LV-shPVT1 groups compared with LV-shNC group of BC cells *in vivo* (Figure 9B). Tumor weight was lessened in LV-shPVT1 groups than LV-shNC groups in vivo (Figure 9C). Tumor growth was slower in LV-shPVT1 groups than LV-shNC groups *in vivo* (Figure 9D). Our experiments revealed that the knockdown of PVT1 could reduce CD44, MET, cyclinD1, CDC20, BCLAF1 and β-catenin expression of BC cells *in vivo* (Figure 9E). IHC experiments proved that the knockdown of PVT1 restrained Ki-67 and BCLAF1 expression (Figure 9F) of BC cells *in vivo*. Our experiments proved that PVT1 facilitated the tumorigenicity of BC via up-regulating BCLAF1.

**Figure 10:**
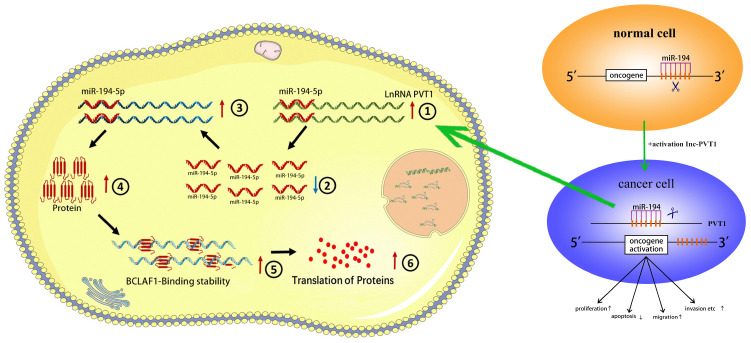
**The schematic diagram of the oncogenic role of PVT1 in BC cells.** PVT1 functions as a miRNA sponge to positively regulate the expression of BCLAF1 through sponging miR-194-5p and subsequently promotes malignant phenotypes of BC cells, and thus it plays an oncogenic role in BC pathogenesis.

As simulated diagram shown as Figure 10, PVT1 acts as a microRNA sponge that actively promotes the expression of BCLAF1 to sponge miR-194-5p and subsequently increases malignant phenotypes of BC cells. PVT1 is dramatically upregulated in BC cells and PVT1 could sponge miR-194-5p to closely promote BCLAF1 expression. Up-regulated BCLAF1 protein could facilitate transcription and translation of proteins operating through indispensably abnormal protein signaling pathways, and subsequently accelerate malignant phenotypes of BC cells.

## DISCUSSION

In terms of LncRNAs, transcripts are longer than 200 nucleotides, and lncRNAs are the main members of non-coding RNAs family [14, 17, 21, 23, 28]. Various literature reveal that lncRNAs play vital functions in genes’ regulation, and could regulate biological processes varying in different methods, such as regulating transcription, sponging miRNA, and modifying epigenetic regulation and so on [30–38]. LncRNAs also refer to tumor proliferation, apoptosis, invasion and migration [40, 42, 46, 56, 59], which broaden our knowledge about the biological behavior of diseases especially in bladder carcinomas. Meanwhile, miRNAs are is a kind of classic and potential biomarkers and key regulators in some hot spots included tumor, and play similar roles, including oncogenes, tumor suppressor genes in tumors, regulating the proliferation, migration, apoptosis and other important biological behaviors of tumor cells [17, 18, 20, 23]. Various literatures concluded that lncRNAs were closely related with miRNAs, and our study further confirmed the mutual relationship with them [26, 28, 35–37, 57, 59].

PVT1 is located in chromosome 8q24.21 that is a vulnerable site prone to genetic abnormalities, and it is also the star molecule in the long non-coding RNA family. Its role was demonstrated in other tumors, and has been manifested to be up-regulated in some tumor tissues, including osteosarcoma, squamous carcinoma, lung carcinomas, gastric carcinomas, liver carcinomas, colorectal carcinomas, nasopharyngeal carcinoma and so on [32–37]. PVT1 could involve in the development and progression of cancer, and could play an oncogenic role [38, 41–49]. Nevertheless, the correlation with PVT1 and miR-194-5p was rarely studied in bladder carcinomas.

Various literatures proved the mutual function between lncRNAs and miRNAs [22, 23, 26, 28, 30, 32–37] and our article further described this relationship. LncRNAs act as miRNAs sponges or baits, titrating the miRNAs concentration, thereby restraining miRNAs from binding to specific mRNAs. Numerous literatures have reported the roles of PVT in various tumors. [32–49].

PVT1 regulated oral squamous cell carcinoma by sponging miR-28-5p [34], and non-small cell Lung cancer by sponging miR-17-5p or miR-526b [35, 36], and gastric cancer cells by sponging miR-125 [37], and gallbladder by sponging miR-143. [40], pancreatic ductal adenocarcinoma by sponging miR-20a-5p [45] and so on. Our experiment is consistent with the above researches.

This report elucidated the mutual function between PVT1 and miR-194-5p in bladder carcinomas. Our study revealed that PVT1 sponging miR-194-5p could be the biomarker and therapeutic target for the diagnosis and treatment in the bladder carcinomas.

PVT1 expression was significantly up-regulated in BC samples and cell lines. Most importantly, PVT1 expression was closely related to histological grade and TNM stage in the bladder carcinomas. Our further experiments proved that the down-regulation of PVT1 expression could restrain BC cells proliferation or migration and up-regulate apoptosis and so on. The overexpression of PVT1 accelerated cell proliferation, migration and anti-apoptosis in SV-HUC-1. Our further experiments of the nude mice animals illustrated that the knockdown of PVT1 inhibited tumorigenicity and lessened tumor volume and weight *in vivo*.

LncRNAs could act as sponges to be saturated with miRNAs. However, these different expressions of lncRNAs and miRNAs in various cancers could lead to diverse binding effects. Bioinformatics predicted potential sites to study the mechanism of PVT1 in bladder carcinomas. MiR-194-5p was the underlying targets to be predicted and has been verified. MiR-194-5p was the up-regulation induced by knockdown of PVT1. Moreover, miR-194-5p was down-regulated in BC samples and cell lines. Histological grade and TNM stage were closely related to miR-194-5p expression. MiR-194-5p mimics restrained bladder carcinoma cell progression, however miR-194-5p inhibitor was converse. MiR-194-5p acted as the tumor suppressor gene through regulating BCLAF1 expression. In addition, the knockdown of PVT1 induced miR-194-5p over-expression and dramatically restrained the malignant behaviors of BC cells. Down-regulated miR-194-5p partially rescued the suppression induced by knockdown of PVT1. Our studies displayed that PVT1 could sponge miR-194-5p to participate in the progression of the bladder carcinomas.

As we all known, miRNAs are through the indirect role of protein regulation of life activities and the target genes are regulated by binding to the 3'UTR of specific mRNAs, inducing degradation or transcriptional suppression of the target genes. We predicted potential target genes of miR-194-5p with biological software and explored its mechanism in the bladder carcinomas. Luciferase reporter assay proved that BCLAF1 was the common target gene of miR-194-5p and BCLAF1 played the important role in regulating the evolution of tumors. Various literatures have reported BCLAF1 roles in some carcinomas’ tumorigenesis, such as hepatocellular carcinoma [60] and acute myeloid leukemias tumorigenesis [61].

Up-regulation of miR-194-5p restrained BCLAF1 expression, however, down-regulation of miR-194-5p up-regulated BCLAF1 expression. Therefore, the expression of BCLAF1 was negatively related to miR-194-5p expression. Our studies exhibited that the knockdown of BCLAF1 could reverse malignant BC cells phenotypes promotion induced by PVT1 up-regulation. For example, the knockdown of BCLAF1 dramatically reversed BC cells proliferation and migration promotion induced by PVT1 up-regulation, and clearly reversed BC cells apoptosis suppression induced by PVT1 up-regulation. MiR-194-5p/BCLAF1 axis may be pivotal in the BC cells progression.

In all, our studies manifested that PVT1 downregulation could up-regulate miR-194-5p expression and subsequently restrain BCLAF1 expression as the ceRNA-dependent manner. Furthermore, up-regulated miR-194-5p reversed BCLAF1 expression and BCLAF1 reduction reversed the malignant BC cells phenotypes promotion induced by overexpression of PVT1. The experiments opened up a new thought for novel molecular targets of human carcinomas, particularly in the bladder carcinoma, which broadened our comprehension on lncRNA-miRNA-proteins methods in illness evolution, and provided us an orientation to ulterior explore the disease’s occurrence mechanism, and provide theoretical basis for the new treatment as well. That could be transformed into more fresh and valid remedial method for BC, which could be brought more brilliant prospects for mankind to radically cure BC thus altering the current diagnostic and therapeutic bottleneck for numerous cancer patients, especially advanced BCs in the future.

## CONCLUSIONS

The experiments proved that PVT1 could sponge miR-194-5p to closely promote BCLAF1 expression and subsequently accelerate the malignant BC cells’ phenotypes, and therefore could act as the carcinogene in the BCs. Our experiments could provide some useful directions to further exploration on its pathogenesis of the progression and development of the BC. In conclusion, the experiments revealed that PVT1-miR-194-5p-BCLAF1 axis could play the significant roles in the progression and development of BCs. PVT1 and miR-194-5p are novel and important tumor biomarkers, and could be some underlying diagnostic biomarkers and remedial targets for malignant BC in the future.

## MATERIALS AND METHODS

### Patient specimens

Our study included BC patients who received tumorectomy. We quick-freeze the BC specimens and paired normal peritumoral specimens in liquid nitrogen quickly after resection. Every one patient has signed the written informed consent. The first affiliated hospital of Soochow University research ethics committee approved the experiment.

### Cell lines and cell culture

T24, UM-UC-3 and SV-HUC-1 were obtained from the Institute of Cell Biology, Chinese Academy of Sciences in Shanghai. T24 and UM-UC-3 cells were cultivated in DMEM (Gibco, USA). SV-HUC-1 cells were cultivated in F12 (Life technologies, USA). 1% antibiotics (100 U/ml penicillin and 100 μg /ml streptomycin sulfates) and 10% FBS were blended in DMEM and F12. The atmosphere of the incubator is at 37° C and 5% CO_2_.

### Cell transfection

Specific siRNA oligonucleotides were transiently transfected in T24, UM-UC-3 and SV-HUC-1 cells, si-PVT1 sense (5-CAGCTTCAACCCATTACGATT-3), si-BCLAF1 sense (5-GGTTCACTTCGTATCAGAA), si-NC and si-RNA (si-PVT1, si-BCLAF1) were obtained from Gene Pharma (Suzhou, China). Optimum density BC cells were cultivated and then transfected in 6-well plates. The plasmid vectors (pcDNA3.1-PVT1, negative control) were obtained from Gene Script (Nanjing, China). Cells were cultured for 24 hours before transfection. The cells were then transiently transfected with the corresponding vectors using Lipofectamine 3000 Transfection Reagent based on the product description. After 48 hours, cells transfected with the corresponding vector were collected.

### RT-qPCR

Based on the product descriptions, the whole RNA was extracted from the specimens or the transfected cells using TRIzol reagent (Invitrogen, USA). The cDNA was synthesized from total RNA applying the Prime Script RT Reagent Kit with gDNA Eraser (Takara, Japan). The relative expression levels of PVT1 were measured by RT-qPCR with SYBR Premix Ex Taq II (Takara) on the CFX96 sequence detection system (Bio-Rad). The primer sequences were displayed in Supplementary Table 1. The endogenous controls were Glyceraldehyde 3-phosphate dehydrogenase (GAPDH) and U6 small nuclear RNA. The relative quantification method (2^-ΔΔCt^) was adopted to calculate the expressions that were normalized to endogenous controls.

### Cell proliferation assays

CCK-8 (Beyotime, Shanghai) was applied to cell proliferation based on the product descriptions. Cells were incubated in a 96-well plate for 24 hours and then respectively transfected with siRNAs or plasmids in the CCK-8 assays. 0, 24, 48 and 72 h after transfection, the absorbance in each well was measured at by a microplate reader (Bio-Rad, USA).

### EdU incorporation assay

EdU (Ethynyl-2-deoxyuridine) Apollo DNA in vitro kit (RIBOBIO, Guangzhou) was also used for cell proliferation that was detected by EdU incorporation assay based on the product descriptions. In a word, cells transfected siRNA or plasmid and were incubated with 100 μl of 50 μM EdU per well for 2 h at 37° C, respectively. Cells were visualized under a fluorescence digital camera system.

### Cell migration assay

The cells were cultivated into the 6-well plates in the incubator. About 90% confluence was obtained before the transfection siRNA or plasma transfected cells. Use the sterilization 200ul pet tips to generate clean lines in 6-well plates. Use digital camera system to take photos in each well quickly. A day later, the picture was taken again. The travel distance is set at 0 and 24 hours.

### Flow cytometry assay

SiRNAs or plasmid vectors were respectively transfected in bladder T24, UM-UC-3 and SV-HUC-1 cells. For 48 hours after transfection, cells were collected and resuspended in fixation fluid 5 μl of Annexin V-FIFC and 10 μl of propidium iodide were added to 195 μl of cell suspension. Flow cytometry (Beckman, USA) was applied to detect cell apoptosis.

### Western blot analysis

Protein was separated by 10% SDS–PAGE and transferred to PVDF membranes. After blocking in the 5% non-fat milk and the membranes were incubated overnight for at least 16h in 4° C with the primary antibody. At room temperature for 1-2h, the membranes were then incubated with a secondary antibody and enhanced chemiluminescence ECL kit (Beyotime Biotechnology, China) was visualized. β-actin was the internal standard, and the antibodies were demonstrated in Supplementary Table 2.

### Dual-luciferase assays

Based on the product descriptions, dual-Luciferase Reporter Assay System (Promega, USA) was used for the Dual-luciferase reporter assays. The binding and mutant sequences were respectively cloned into pmirGLO Dual-luciferase vectors (Fubio Biological Technology Co, Suzhou, China). PVT1, BCLAF1 WT or Mut constructed and co-transfected along with miR-194-5p mimics or NC, and then transfected with Lipofectamine 3000 and incubated for 48 h. Microplate reader was applied to measure the luciferase activities.

### Mouse model experiments

Our experiment was approved by Institutional Ethics Review Board of Soochow University. 5-wk old male BALB/c nude mice were divided into two groups and each group included 5 mice. LV-PVT1 and LV-NC were purchased from Genechem (Shanghai, China). 2×10^6^ UM-UC-3 cells were injected into the mice dorsal flank regions. Every 5 days, tumor growth was measured. The formula, a*b^2^/2 (a represents the long diameter and b refers to the short diameter), was used to calculate tumor volume. Mice were executed after 30 days and the subcutaneous weight of each tumor was measured.

### Statistical analysis

Assays were performed in triplicate at least and data were shown as mean ± standard deviation (SD) of those biological replicates or samples. SPSS 20.0 software (IBM, Chicago, IL, USA) etc. were used to analyze assays’ statistical analyses. Paired samples t-test was used to analyze the relative expression of PVT1 and miR-194-5p. ANOVA was used to analyze CCK-8 assay data. The independent samples t-test was used to analyze other data. P< 0.05 was regarded as the statistically significant one.

## Supplementary Material

Supplementary Tables
