# Correction for: LncRNA PVT1 accelerates malignant phenotypes of bladder cancer cells by modulating miR-194-5p/BCLAF1 axis as a ceRNA

**DOI:** 10.18632/aging.202683

**Published:** 2021-02-14

**Authors:** Mingwei Chen, Rongyuan Zhang, Le Lu, Jian Du, Chunyang Chen, Keke Ding, Xuedong Wei, Guangbo Zhang, Yuhua Huang, Jianquan Hou

**Affiliations:** 1 Department of Urology, The First Affiliated Hospital of Soochow University, Suzhou 215006, Jiangsu Province, China; 2 Department of Urology, The Fourth Affiliated Hospital, Zhejiang University School of Medicine, Yiwu 322000, Zhejiang Province, China; 3 Jiangsu Institute of Clinical Immunology, The First Affiliated Hospital of Soochow University, Jiangsu Key Laboratory of Clinical Immunology, Soochow University, Jiangsu Key Laboratory of Gastrointestinal Tumor Immunology, Suzhou 215006, Jiangsu Province, China

**Keywords:** correction

Original article: Aging. 2020; 12:22291–22312.  . https://doi.org/10.18632/aging.202203

**This article has been corrected:** The authors replaced Panel **A** in **Figure 7**, because images of PCDNA3.1-PVT1 + si-NC group were misplaced and now were corrected with the corresponding images from the same set of experiments. Panel **C** in **Figure 8** (the 0H scratch image of miR-194-5p inhibition group) was also misplaced in the previous version of the figure, and now it is replaced with the correct image from the original set of experiments. This alteration does not affect the results or conclusions of this work. The new **Figure 7** and **Figure 8** are presented below.

**Figure 7:**
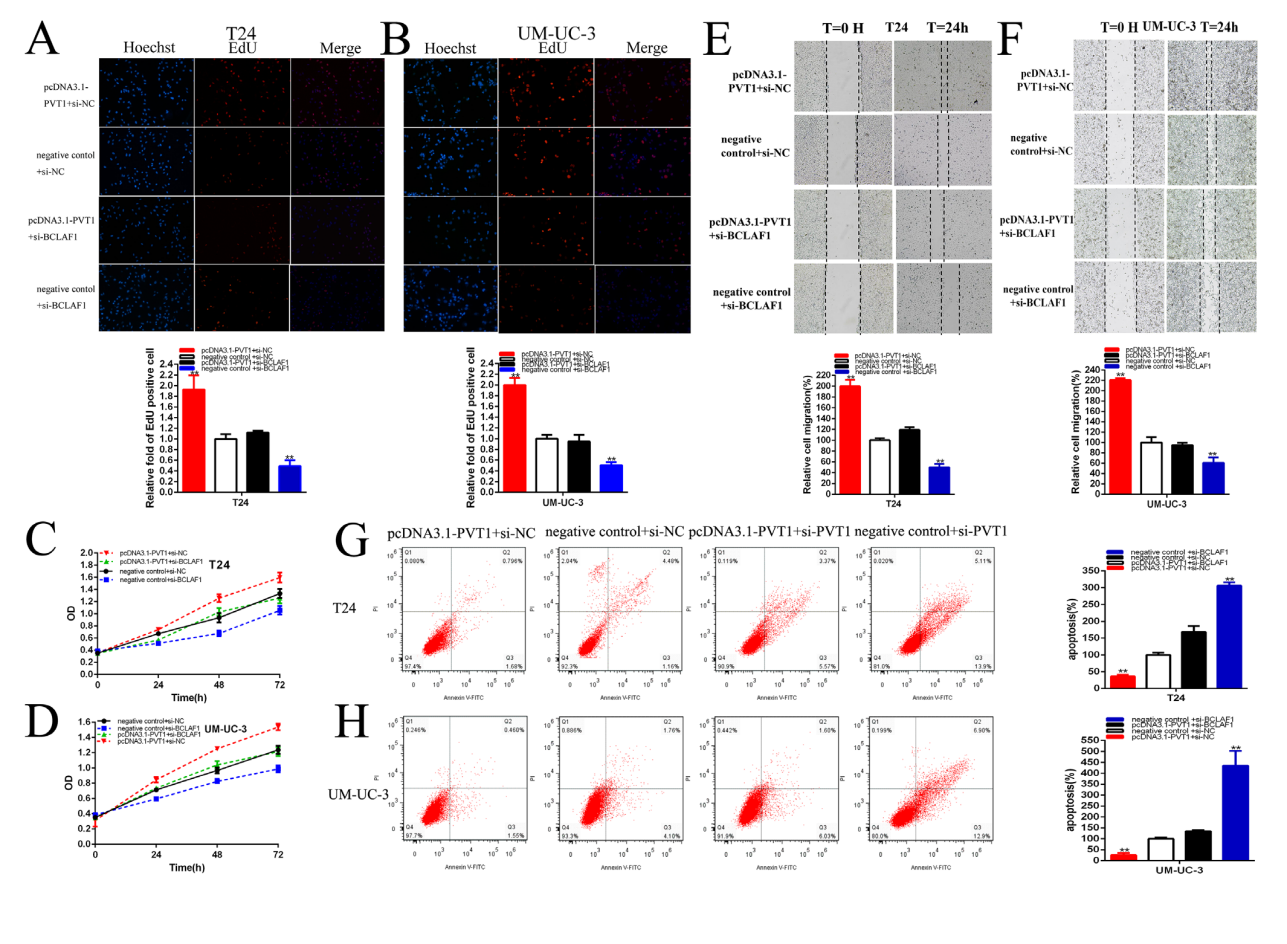
**PVT1 positively regulates BCLAF1 expression via sponging miR-194-5p.** Knockdown BCLAF1 significantly reversed cell proliferation promotion induced by Overexpression PVT1 (EdU, **A, B,** CCK8, **C, D**). Knockdown BCLAF1 significantly reversed cell migration promotion induced by overexpression PVT1 (**E, F**). Knockdown BCLAF1 significantly reversed cell apoptosis inhibition induced by overexpression PVT1 (**G, H**). (*P < 0.05, **P < 0.01).

**Figure 8:**
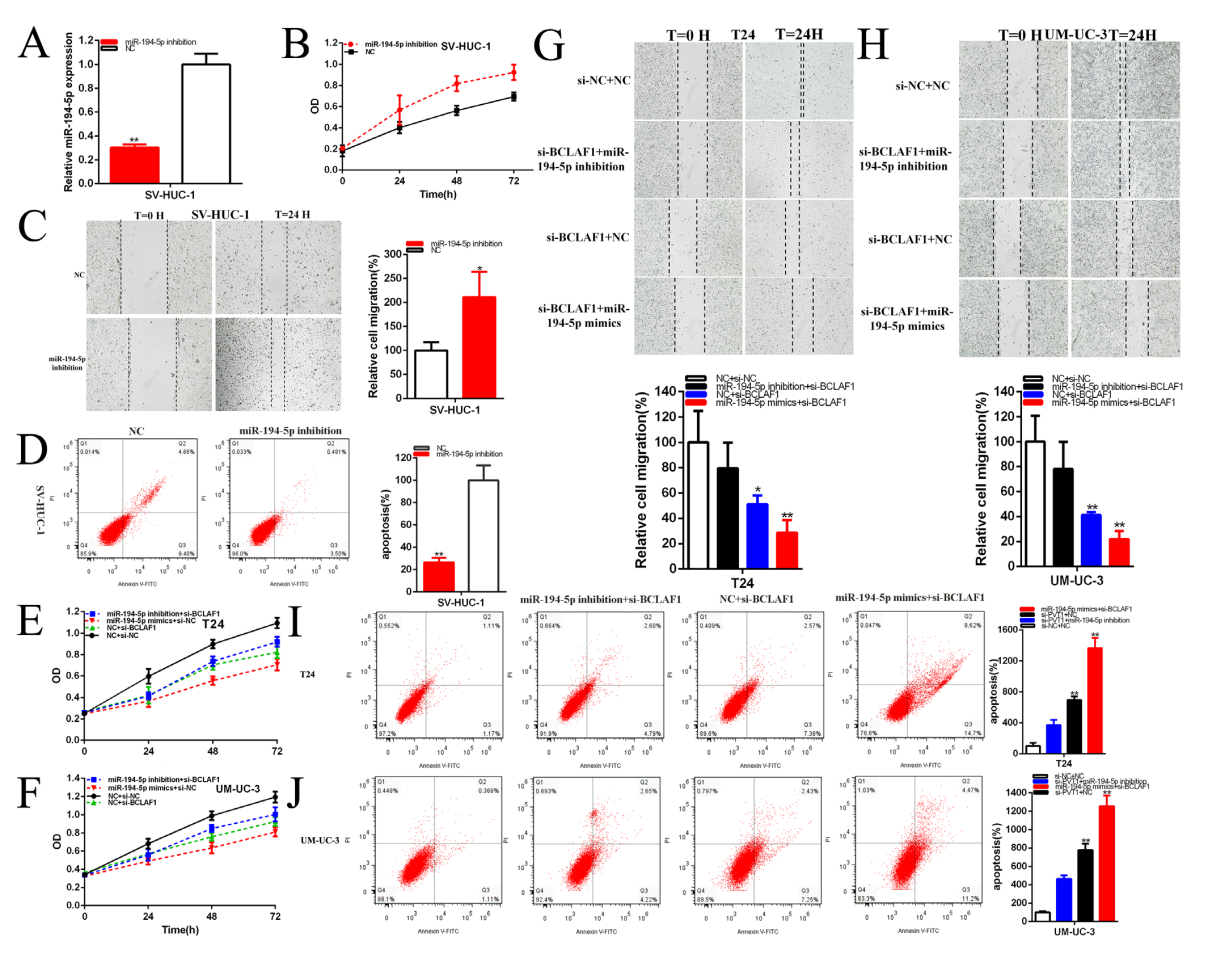
**MiR-194-5p positively regulates BCLAF1 expression.** The relative expression level of miR-194-5p was reduced by miR-194-5p inhibitor in SV-HUC-1 (**A**). Cell proliferation was detected in SV-HUC-1 after transfection of miR-194-5p inhibitor (**B**). The relative cell migration was accelerated after transfection of miR-194-5p inhibitor in the SV-HUC-1 (**C**). Apoptotic cells were measured after transfection of miR-194-5p inhibitor in SV-HUC-1 (**D**). Cell proliferation was detected in both bladder carcinomas cell lines after co-transfection with si-NC+NC and si-BCLAF1+miR-194-5p inhibitor or mimics (**E**, **F**). The relative cell migration after co-transfection with si-NC+NC, si-BCLAF1+miR-194-5p inhibitor or mimics, and the representative images were as follow (**G**, **H**). The apoptotic cells were measured after co-transfection with si-C+NC, si-BCLAF1+miR-194-5p inhibitor or mimics by flow cytometry analysis (**I**, **J**). (*P < 0.05, **P < 0.01).

