# Supplementary material for: LncRNA PVT1 accelerates malignant phenotypes of bladder cancer cells by modulating miR-194-5p/BCLAF1 axis as a ceRNA
Source: Aging (Albany NY). 2020 Nov 16;12(21):22291–312. doi: 10.18632/aging.202203 (PMC7695393; doi:10.18632/aging.202203)
Supplement: Supplementary Tables [file aging-12-202203-s001..pdf]

SUPPLEMENTARY TABLES

Supplementary Table 1. The primers for real-time QPCR.

Gene	Forward or Reverse	Primer sequence
PVT1	Forward	5'- GCCCCTTCTATGGGAATCACTA -3'
	Reverse	5'- GGGGCAGAGATGAAATCGTAAT -3'
BCLAF1	Forward	5'- ATGAGACGACCTTATGGGTACA -3'
	Reverse	5'- AGAGTGCCTTCTATTCCAGACAG -3'
miR-194-5p	Forward	5'- GCCGCTGTAACAGCAACTCCAT -3'
	Reverse	5'- GTGCAGGGTCCGAGGT -3'
GAPDH	Forward	5'- CGCTCTCTGCTCCTCCTGTTC-3'
	Reverse	5'-ATCCGTTGACTCCGACCTTCAC-3'
U6	Forward	5'- CTCGCTTCGGCAGCACA-3'
	Reverse	5'- ACGCTTCACGAATTTGCGT-3

BCLAF1: BCL2 associated transcription factor 1.

Supplementary Table 2. Antibodies used for western blots.

Antibody	Description	Dilution	Supplier	Country
Anti-CDC20	Mouse monoclonal	1:1000	Santa Cruz	USA
Anti- CD44	Mouse monoclonal	1:1000	Santa Cruz	USA
Anti-Met	Mouse monoclonal	1:1000	Santa Cruz	USA
Anti-β-catenin	Mouse monoclonal	1: 1000	Santa Cruz	USA
Anti-Cyclin D1	Rabbit monoclonal	1: 1000	Beyotime	China
Anti-BCLAF1	Rabbit polyclonal	1: 500	BBI	China
Anti-BCLAF1	Mouse monoclonal	1: 500	Santa Cruz	USA
Anti-β-Actin	Mouse monoclonal	1: 1000	Beyotime	China
Anti-BCLAF1	Rabbit Polyclonal	1: 500	Proteintech	USA

CDC20: cell division cycle 20; BCLAF1: BCL2 associated transcription factor 1; CD44: CD44 molecule; Met: MET proto-oncogene, receptor tyrosine kinase; β-catenin: catenin beta 1.
